# Unprecedented Use of NHC Gold (I) Complexes as Catalysts for the Selective Oxidation of Ethane to Acetic Acid

**DOI:** 10.3390/ma14154294

**Published:** 2021-07-31

**Authors:** Ana P. C. Ribeiro, Inês A. S. Matias, Poorya Zargaran, A. Stephen K. Hashmi, Luísa M. D. R. S. Martins

**Affiliations:** 1 Centro de Química Estrutural and Departamento de Engenharia Química, Instituto Superior Técnico, Universidade de Lisboa, Av. Rovisco Pais, 1049-001 Lisboa, Portugal; ines.matias@tecnico.ulisboa.pt; 2 Organisch-Chemisches Institut, Heidelberg University, Im Neuenheimer Feld 270, 69120 Heidelberg, Germany; poorya.zargaran@gmail.com (P.Z.); hashmi@hashmi.de (A.S.K.H.); 3 Chemistry Department, Faculty of Science, King Abdulaziz University, Jeddah 21589, Saudi Arabia

**Keywords:** NHC gold (I) complex, NHOC gold (I) complex, ethane oxidation, acetic acid, green metrics

## Abstract

The highly efficient eco-friendly synthesis of acetic acid (40% yield) directly from ethane is achieved by the unprecedented use of N-heterocyclic carbene (NHC) and N-heterocyclic oxo-carbene (NHOC) gold(I) catalysts in mild conditions. This is a selective and promising protocol to generate directly acetic acid from ethane, in comparison with the two most used methods: (i) the three-step, capital- and energy-intensive process based on the high-temperature conversion of methane to acetic acid; (ii) the current industrial methanol carbonylation processes, based in iridium and expensive rhodium catalysts. Green metrics determinations highlight the environmental advantages of the new ethane oxidation procedure. Comparison with previous reported published catalysts is performed to highlight the features of this remarkable protocol.

## 1. Introduction

It is well-recognized that N-heterocyclic carbene (NHC) ligands have played an increasingly significant role in designing homogeneous catalysts. NHC metal complexes are able to catalyze different types of reactions, such as alkene activation, alkyne hydration, hydroamination, hydrosilylation or C–C couplings [1,2,3,4,5].

Among the most challenging chemical reactions are alkane oxidations, in particular the oxidation of the light gaseous ones [6,7]. In fact, despite alkanes being the most abundant and relatively low-cost form of carbon, their inertness has hampered their use as feedstocks for the synthesis of functionalized added-value products, namely, carboxylic acids. Harsh reaction conditions (high temperatures, acidic medium, long reaction times, etc.) and low product yields and/or selectivities are limitations that should be overcome through the finding of efficient catalytic systems for the selective activation of the C−H bonds.

Acetic acid, a large tonnage commodity of high importance in view of its wide industrial use (the global acetic acid market reached a volume of 9.07 million tons in 2020) [8], is mainly produced via a three-step, capital- and energy-intensive process based on the high-temperature conversion of methane or coal to syn-gas, conversion of syn-gas to methanol and finally methanol carbonylation to acetic acid [9]. The current industrial methanol carbonylation processes are CATIVA™ [10], and MONSANTO™ [11], based in iridium and expensive rhodium catalysts, respectively. They have benefits such as high yield of the product, but the disadvantages are also apparent, which include the environmental ones as well as the severe corrosion to equipment by the required iodide co-catalyst.

Acetic acid may also be derived from acetaldehyde (at 150–160 °C and 80 bar) over either cobalt or manganese acetate [12], or by the halide-free carbonylation of dimethyl ether over zeolite catalysts [13]. The latter selectively (>99%) [14,15] yields methyl acetate which undergoes hydrolysis to methanol and acetic acid. Although promising, currently reaction rates do not meet commercial targets.

Therefore, being able to synthesize acetic acid directly by oxidation of an alkane would be much more clean, cheaper and therefore wiser, but also challenging. A suitable alkane for such a process is ethane, although some advances on the production via carboxylation with CO of acetic acid from methane have been reported [16,17,18,19,20]. However, the high stability of the C–H bond in ethane has severely hindered the development of a viable process for the partial oxidation of ethane under mild conditions. Adding further complication by the fact that one must not only activate the inert alkane and be able to subdue the oxidation of desirable products into compounds such as formic acid or carbon dioxide.

**Scheme 1:**
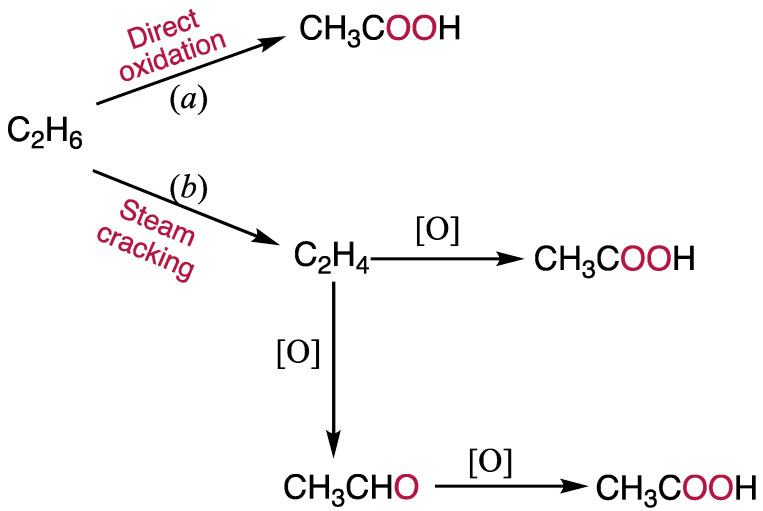
Direct and indirect ways to obtain acetic acid from ethane.

Direct (Scheme 1*a*) [21,22,23] or indirect (Scheme 1*b*) [23] processes from ethane have been considered. The latter first requires steam cracking of ethane to ethene (in order to activate the alkane substrate), followed by a two-step ethene-acetaldehyde-acetic acid process. Although studies have shown such routes can lead to economic equivalence with methanol carbonylation (at comparable levels of production scale) [23], direct oxidation of ethane is always preferable as it annuls the need for the isolation of intermediate steps.

A wide range of catalysts and oxidant agents have been tested in the partial oxidation of ethane to acetic acid, either in homo- or heterogeneous conditions [23]. Vital contributions are iridium cluster catalysts reported [24] by Ma et al., exhibiting notable performance for the selective oxidation of ethane by molecular oxygen at 100 °C or the catalytic conversion of ethane to acetaldehyde on an inert gold electrode achieved by Takehira et al. [25]. However, none of the tested catalytic systems have demonstrated the required performance for industrial commercialization. This includes the only gold catalyst reported, HAuCl_4_, which, with H_2_O_2_ in water at 90 °C for 1 h yielded CH_3_CHO, EtOH and CO_2_, but no traces of acetic acid.

T. Strassner et al., reported [26] (to the best of our knowledge) the only example of application of NHC-complexes as catalysts for C–H activation. They used palladium bis-carbene complexes with potassium peroxodisulfate in trifluoroacetic acid to convert methane into the methyl ester of trifluoroacetic acid. However, other substrates were not tested. In the current study we successfully explored its diagonally adjacent element, gold. Therefore, the unprecedented use of gold complexes as pre-catalysts for the direct oxidation of ethane to acetic acid (Scheme 1*a*), under environmentally friendly conditions (e.g., aqueous medium, low temperature) is disclosed herein.

## 2. Materials and Methods

**Scheme 2:**
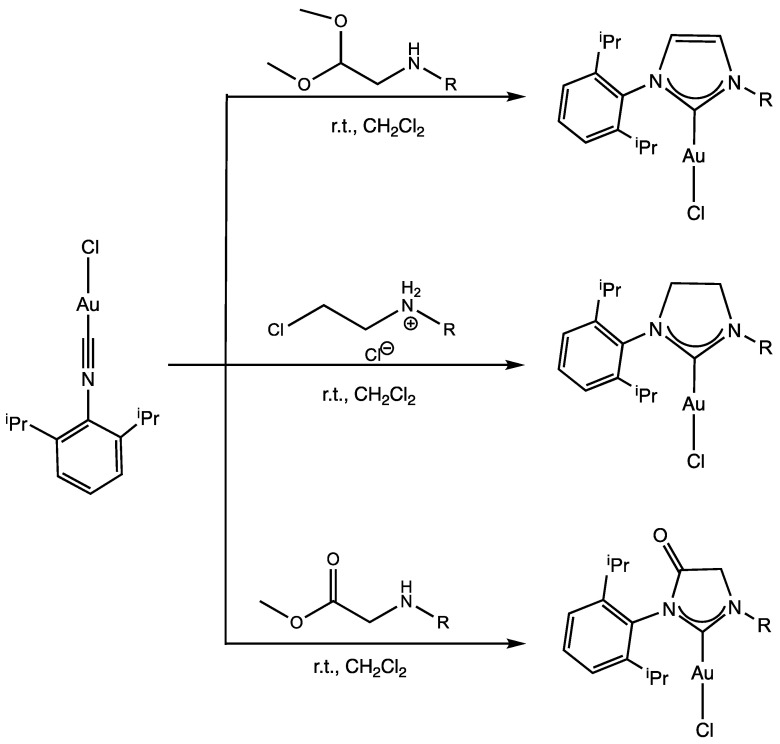
Synthesis of N-heterocyclic carbene (NHC) and N-heterocyclic oxo-carbene (NHOC) gold(I) complexes **1**–**4**, R = cyclohexyl (**1**–**3**) or cyclododecyl (**4**). Adapted from: Zargaran, P et al., Adv. Synth. Catal.; published by Wiley, 2018.

All solvents and reagents were obtained from commercial sources (Sigma-Aldrich, Munich, Germany) and were used without further purification. N-heterocyclic carbene (NHC) and N-heterocyclic oxo-carbene (NHOC) gold(I) complexes **1**–**4** were synthetized according to the literature [27]. Briefly, the strategic synthesis encompasses isonitrile-based routes as depicted in Scheme 2.

The catalytic oxidation of ethane to acetic acid was performed in a stainless-steel reactor with a capacity of 13.5 mL. Into the reactor, 4.33 mmol of oxidant (K_2_S_2_O_8_) and 1.50 mol of catalyst (**1**–**4**) were added to 6 mL of (1:1) H_2_O/CH_3_CN (or 5.5 mL of trifluoroacetic acid). When appropriate, the radical trap (TEMPO) was added. The reactor was closed and flushed three times with ethane before being pressurized to 3 atm (0.78 mmol). The mixture was heated in an oil bath at 80 °C (higher temperatures are not recommended for this reaction due to a decrease of ethane solubility and to the decomposition of the oxidant) [28], for 20 h. After the reaction, the reactor was placed in an ice bath to cool down to room temperature, degassed and opened. 5 mL of diethyl ether (to extract organic products and precipitate the catalyst) and 90 μL de *n*-butyric acid (as internal standard) was added to 1 mL of reaction and let stirring for 30 min. Then, the mixture was filtrated and analysed by gas chromatography (GC), using the internal standard method. GC analyses were carried out at a FISONS Instruments GC 8000 (Tokyo, Japan) series gas chromatograph with a FID detector and a capillary column (DB-WAX 0.32 mm and 30 m of internal diameter and column length respectively using helium as the carrier gas. The software was the Jasco-Borwin v.1.50. (Tokyo, Japan) The temperature of injection was 240 °C. The starting temperature is 100 °C for 1 min, then increases 10 °C/min until 180 °C and is kept at this temperature for 1 min. Each value of yield results from the average obtained from runs with identical results.

## 3. Results and Discussion

### 3.1. Direct Oxidation of Ethane to Acetic Acid

**Figure 1:**
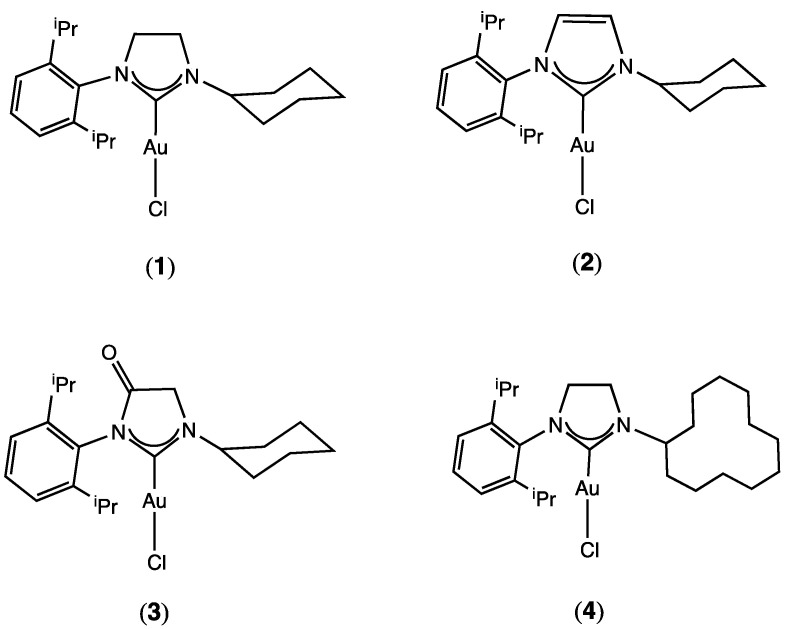
Structures of five-membered saturated (**1** and **4**) or unsaturated (**2**) N-heterocyclic carbene (NHC) gold(I) chloride complexes as well as of an unsymmetrical N-heterocyclic oxo-carbene (NHOC) gold(I) chloride complex (**3**).

Five-membered saturated or unsaturated N-heterocyclic carbene (NHC) and unsymmetrical N-heterocyclic oxo-carbene (NHOC) gold(I) chloride complexes were synthesized [27]. Aiming to investigate structure-reactivity relationships, a set of cyclic carbene complexes was selected having on one nitrogen atom the 2,6-diisopropylphenyl group and on the other nitrogen atom the cyclohexyl or the cyclododecyl group (Figure 1). The monodentate NHC electron rich σ-donor ligands are known to strongly bind to Au(I) in a “push-pull” mechanism [29].

The direct oxidation of ethane to acetic acid (Scheme 1*a*) was performed using potassium peroxodisulfate as oxidant and Au(I) complexes **1–4** as catalysts in aq. acetonitrile at 80 °C for 20 h (optimized conditions). The effect of the structure of the N-heterocycle as well as the substitution pattern at the nitrogen atoms were analyzed under exactly the same reaction conditions.

**Figure 2:**
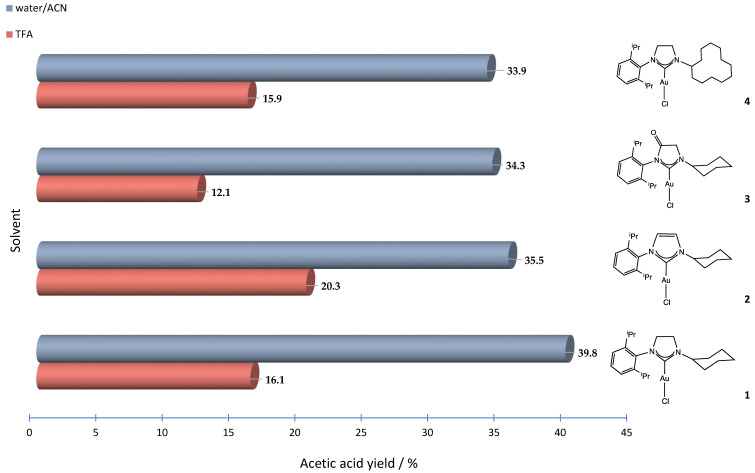
Acetic acid yields achieved by direct oxidation of ethane by K_2_S_2_O_8_, catalysed by complexes **1**–**4**.

All the gold(I) catalysts **1**–**4** performed with remarkable selectivity (acetic acid was the only product detected by chromatographic analysis) and high activity (Table 1 and Figure 2). The saturated NHC complex **1** bearing the cyclohexyl substituent at the nitrogen atom exhibited the highest ethane conversion (acetic acid yield of 40%) while the effect of different ring size substituent of complexes **1** and **4** was moderately significant (15% yield decrease from a C_6_ to C_12_ ring, Figure 2). Moreover, the saturated NHC Au(I) complex **1** delivered higher ethane conversion compared to complex **2** with different imidazoline structure but same side chains. An almost similar behavior was observed for the unsaturated gold(I) complex **2** (36% yield of acetic acid) and the NHOC gold(I) complex **3** which showed a slightly low conversion (acetic acid yield of 34%).

The observed trend regarding the structure of the central cycle is in accordance with the behavior found previously for other catalytic reactions of these complexes [27]. The higher electron density at the metal center of the saturated NHC ligand compared with the unsaturated one, in addition to a synergetic effect of π back-donation and σ donation of the saturated NHC ligand, leads to a more stable complex and this stability plays an important role in the catalytic properties of the gold(I) complexes, in particular for long reactions.

Blank experiments were performed in the presence of C_2_H_6_ and K_2_S_2_O_8_ (metal-free as analyzed by ICP) and confirmed (see entry 5 of Table 1 for catalyst **1**) that almost no acetic acid formation was detected unless the Au catalyst was used.

The traditional solvent for the oxidation of ethane, trifluoroacetic acid (TFA), [22,30,31,32] was also used with the Au(I) complexes **1**–**4**. As depicted in Figure 2, the performance of all pre-catalysts is much worse in TFA, leading to yield decreases of ca. 60% when compared with the results attained in the water/acetonitrile (1:1) mixture.

**Scheme 3:**
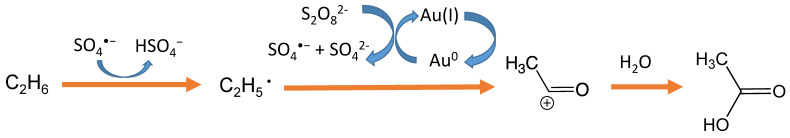
Proposed reaction mechanism for the oxidation of ethane to acetic acid.

A free radical mechanism for the oxidation of ethane to acetic acid, involving acyl radical formation, oxidation and subsequent hydroxylation by water (Scheme 3), is envisaged from the performed radical trap experiments. In fact, when (2,2,6,6-tetramethylpiperidin-1-yl)oxyl (TEMPO) radical was used as an oxygen radical trap [19], a drastic suppression of the oxidation reaction and consequent acetic acid yield decrease, from 39.8% to 13.4%, was detected (compare entries 1 and 7 of Table 1).

To date, most of the oxidations of ethane to acetic acid were obtained as side reactions of ethane carboxylation, with carbon monoxide, to propionic acid in the presence of K_2_S_2_O_8_ and in trifluoroacetic acid (TFA) at 80 °C. Acetic acid is formed as a result of the competitive metal catalyzed partial oxidation of ethane by the peroxodisulfate salt, which led the researchers to run the reaction in the absence of CO [19,21,22,30], thus using the peroxodisulfate salt as oxidizing agent and trifluoroacetic acid as the solvent and carboxylating agent. These results are presented in Table 2 in order to have an insight on the catalytic performance of **1**–**4** in our procedure compared with the state of art for oxidation of ethane.

Even in non-optimized reaction conditions (the water/acetonitrile mixture proved to lead to superior performance, Figure 2), our gold pre-catalysts **1**–**4** exhibited, in general, higher selectivity and acetic acid yields for a much lower catalyst load than the previously tested complexes [22,30].

It is also worth to highlight the remarkable performance of the NHC gold pre-catalysts **1**–**4**, in particular of complex **1**, which led to the same (40%) acetic acid yield that was previously obtained by the best catalyst so far reported [22] for the oxidation of ethane, [ReClF{N_2_C(O)Ph}(Hpz)_2_(PPh_3_)], but under highly intensive reaction conditions (e.g., catalyst load ca. 13 times higher and much more hazardous medium).

### 3.2. Green Metrics

Aiming at evaluating, as much quantitative as possible, the improvement brought by our new ethane oxidation procedure, several dedicated green analytical chemistry metrics were calculated and are presented in Table 3 [32,33,34,35,36].

The Environmental factor (E-factor), total weight (in kg) of all waste generated in an industrial process per kilogram of product [33], was calculated to expose the waste of the present process. An E-factor value approaching zero means that less waste is generated and, therefore, the process exhibits higher environmental sustainability. As shown in Table 3, the use of the solvent mixture H_2_O/acetonitrile ACN led always to lower E-factor values than the ones obtained under the same reactional conditions but using TFA as solvent. Although without a direct quantification of the hazards or the environmental risks of the produced waste, the decrease of the values for the E-factor parameter highlights the change for a greener solvent system. A theoretical E-factor was also estimated under the assumption of a complete conversion of reagents and a minimization of waste, and we can observe (Table 3) that this process, per se, is highly promising as a technological or industrial process with a small environmental impact. For catalyst **1** in water/ACN mixture, entry 1 of Table 3, corresponding to the highest attained acetic acid yield (Table 1 and Figure 2), the difference between the E-factor and the theoretical E-factor values is the smallest, being also smaller than the one for pre-catalyst **1** in TFA (entry 2, Table 3). As expected, a low number of generated products indicates a low activity process with room for bigger improvement.

Following a multi-metrics approach, other parameters were determined. Atom Economy (AE) and Mass Intensity (MI) provide important indicators of the limitations of a chemical process, since AE quantifies the atomic efficiency of the catalytic process and MI its applicability (as quantifies reaction efficiency, stoichiometry, amount of solvents, all reagents and auxiliary substances) [34,35]. Thus, an increase in AE should lead to a decrease in MI. In this work, the atom economy is 20% (see Table 3). This value may appear low, but we should note that oxidation reactions usually use a huge excess of oxidant (this new process uses a reagent:oxidant ratio of 1:6). A comparison of our results, namely for complex **1** (entry 1 of Table 3), with the previous complex allowing to achieve the nearest yield of acetic acid (40.9%), i.e., [ReClF{N_2_C(O)Ph}(Hpz)_2_(PPh_3_)] (Hpz = pyrazole) [22] (entry 7 of Table S1, ESI), shows that the value of MI doubles for our new process (as the initial amount of reagents in the method using the Re catalyst is almost the double). This is an evidence of the importance of a reaction input in the desired results.

Metrics considering the process upscaling possibility, such as *Reaction Mass Efficiency* (*RME*) and *Atom Utilization* (*AU*) were also considered. *RME* is inversely related to the overall *E-factor* [33] (Table 2), being only slightly higher for our catalytic method than for processes currently known to possess excellent *E-factor* values [34], as it is a by-products-free method. This is also reflected by the *AU* values (Table 3), since all the components of the reaction are used to produce the desired acetic acid, minimizing waste production.

To help in the quantification of the “greenness’’ of the overall process, a metric designated Solvent and Catalyst Environmental Impact Parameter (f), which takes into account the real masses of materials used in the process (and if they are recycled, recovered or eliminated) was determined (Table 3). Several reasons can lead to this improvement, namely the higher selectivity (thus, higher efficiency) of our Au(I) catalysts. In addition, the amount of gold complexes used by us is significantly lower (1.5 vs. 20 μmol, see Table S1, ESI), indicating that, per atom, the conversion proceeds in a more efficient way. This seems to be the case for complex **1** that, accordingly, should be more stable than the rhenium catalyst (entry 7, Table S1, ESI).

## 4. Conclusions

An efficient and simple catalytic method for the acetic acid synthesis via the direct oxidation of ethane has been developed. In view of the encouraging results achieved by using NHC gold(I) catalysts, also attested by the determined green metrics, a better understanding of the mechanism involved as well as further studies involving optimization (e.g., catalyst recycling) is underway to help the design and growth of a sustainable catalytic process for this pivotal transformation.

## Figures and Tables

**Table 1:** Direct oxidation of ethane by K_2_S_2_O_8_ in water/acetonitrile (1:1) mixture.

Entry	Catalyst	C_2_H_6_	Catalyst Amount (μmol)	Yield (%) ^a^		TON ^b^	
		(atm)		Acetic Acid	Propionic Acid	Acetic Acid	Propionic Acid
1	**1**	3	1.5	39.8		210	
2	**2**	3	1.5	35.5		187	
3	**3**	3	1.5	34.3		181	
4	**4**	3	1.5	33.9		178	
5		3		2.2			
6 ^c^	**1**	3	1.5				
7 ^d^	**1**	3	1.5	13.4		67	

Reaction conditions: water/acetonitrile (1:1) mixture as solvent (6 mL), K_2_S_2_O_8_ as oxidant, T = 80 °C, 20 h. ^a^ Molar yield (%) based on C_2_H_6_, i.e., moles of acid per 100 mol of ethane. ^b^ TON (turnover number) = moles of product per mol of catalyst. ^c^ no oxidant. ^d^ in the presence of TEMPO.

**Table 2:** Comparison of the catalytic performance of Au(I) complexes **1–4** and the state of the art for the oxidation of ethane.

Entry	Catalyst	C_2_H_6_	Catalyst Amount (μmol)	Yield (%) ^a^		TON ^b^		Ref.
		(atm)		Acetic Acid	Propionic Acid	Acetic Acid	Propionic Acid	
1	**1**	3	1.5	16.1		18		this work
2	**2**	3	1.5	20.3		22		
3	**3**	3	1.5	12.1		13		
4	**4**	3	1.5	15.9		18		
5	[ReCl_2_{κ^2^-N,O-N_2_C(O)Ph}(PPh_3_)_2_]	3	20	3.3		2		22
6	[ReCl_2_{N_2_C(O)Ph}(Hpz)(PPh_3_)_2_]	3	20	3.7		7		
7	[ReCl_2_{N_2_C(O)Ph}(Hpz)_2_(PPh_3_)]	3	10	24.1	0.7	19	0.6	
8	[ReOCl_3_(PPh_3_)_2_]	3	20	14.0	3.0	6	1.4	
9	[ReCl_3_{κ^3^-HC(pz)_3_}]	3	20	5.1		2		
10	[ReO_3_{κ^3^-SO_3_C(pz)_3_}]	3	20	27.9	1.0	13	0.4	
12	[ReClF{N_2_C(O)Ph}(Hpz)_2_(PPh_3_)]	5	20	40.9	5.4	31	4.0	
13	[ReOCl_2_(C_5_H_4_N(CO*O*))(PPh_3_)]	10	20	5.0	1.3	7	2.9	30
14	Na_2_[MoO_4_]	3	20	25.0		63		31
15	Nb_2_O_5_	1.5	20	18.0		10		
16	H_4_[PMo_11_VO_40_]·34H_2_O	5	2.5	36.3	2.8	222	17	19
17	H_5_[PMo_10_V_2_O_40_]·32H_2_O	5	2.5	20.2	3.2	124	20	
18	H_6_[PMo_9_V_3_O_40_]·34H_2_O	5	2.5	35.2	2.9	216	18	

Reaction conditions: TFA as solvent, K_2_S_2_O_8_ as oxidant, T = 80 °C, 20 h. ^a^ Molar yield (%) based on C_2_H_6_, i.e., moles of acid per 100 mol of ethane. ^b^ TON (turnover number) = moles of product per mol of catalyst.

**Table 3:** Green chemistry metrics for the oxidation of ethane to acetic acid catalyzed by Au(I) complexes **1–4**.

Entry	Solvent	Catalyst	Theoretical *E-Factor*	*E-Factor*	*AE* (%)	*MI*	*RME*	*AU* (%)	*f*
1	H_2_O/ACN	**1**	0.2	0.5	20.0	36.5	66.5	100	107.1
2	TFA			2.7		90.3	26.9		696.8
3	H_2_O/ACN	**2**	0.2	0.7	20.0	41.0	59.4	100	120.1
4	TFA			1.9		71.6	34.0		552.6
5	H_2_O/ACN	**3**	0.2	0.8	20.0	42.4	57.1	100	124.3
6	TFA			4.0		120.2	20.1		927.2
7	H_2_O/ACN	**4**	0.2	0.8	20.0	42.9	55.3	100	125.8
8	TFA			2.9		91.6	25.9		705.7

## Data Availability

Data is contained within the article or supplementary material.

## Supplementary Materials

The following are available online at https://www.mdpi.com/article/10.3390/ma14154294/s1, Table S1: Examples of green chemistry metrics applied in this work, Table S2: Calculated metrics for the oxidation of ethane to acetic acid, Table S3: Calculated frequency metrics for the oxidation of ethane to acetic acid.
