# Supplementary material for: Unprecedented Use of NHC Gold (I) Complexes as Catalysts for the Selective Oxidation of Ethane to Acetic Acid
Source: Materials (Basel). 2021 Jul 31;14(15):4294. doi: 10.3390/ma14154294 (PMC8347251; doi:10.3390/ma14154294)
Supplement: Supplementary file 1 [file materials-14-04294-s001.zip › materials-1277890-supplementary.pdf]

materials

MDPI

Supplementary

Unprecedented Use of NHC Gold (I) Complexes as Catalysts for the Selective Oxidation of Ethane to Acetic Acid

Ana P.C. Ribeiro [1,*], Inês A.S. Matias [1], Poorya Zargaran [2], A. Stephen K. Hashmi [2,3] and Luísa M.D.R.S. Martins [1,*]

[1] Centro de Química Estrutural and Departamento de Engenharia Química, Instituto Superior Técnico, Universidade de Lisboa, Av. Rovisco Pais, 1049-001 Lisboa, Portugal; ines.matias@tecnico.ulisboa.pt
[2] Organisch-Chemisches Institut, Heidelberg University, Im Neuenheimer Feld 270, 69120 Heidelberg, Germany; poorya.zargaran@gmail.com (P.Z.); hashmi@hashmi.de (A.S.K.H.)
[3] Chemistry Department, Faculty of Science, King Abdulaziz University, Jeddah 21589, Saudi Arabia
* Correspondence: apribeiro@tecnico.ulisboa.pt (A.P.C.R.); luisammartins@tecnico.ulisboa.pt (L.M.D.R.S.M.); Tel.: +35-12-1841-9389 (L.M.D.R.S.M.)

Citation: Ribeiro, A.P.C.; Matias, I.A.S.; Zargaran, P.; Hashmi, S.K.; Martins, L.M.D.R.S. Unprecedented Use of NHC Gold(I) Complexes as Catalysts for the Selective Oxidation of Ethane to Acetic Acid. *Materials* **2021**, *14*, 4294 . https://doi.org/10.3390/ma14154294

Academic Editors: Josep Puig-martí-Luis and Lucia Carlucci

Received: 12 June 2021
Accepted: 28 July 2021
Published: 31 July 2021

Publisher's Note: MDPI stays neutral with regard to jurisdictional claims in published maps and institutional affiliations.

Copyright: © 2021 by the authors. Submitted for possible open access publication under the terms and conditions of the Creative Commons Attribution (CC BY) license (http://creativecommons.org/licenses/by/4.0/).

Table S1. Examples of green chemistry metrics applied in this work.

Parameter	Formula
E- Factor	$E = \frac{\text{total weight of all waste generated in technological or industrial process}}{\text{per kilogram of a product}}$
Atom Economy (AE)	$AE(\%) = \frac{\sum Molecular\ Weigth\ of\ the\ products}{\sum Molecular\ Weigth\ of\ the\ reagents} \times 100$
**Atom Efficiency (AEf)**	$AEf = AE(\%) \times Yield$

Mass Intensity (MI)	$$\mathrm{MI} = \frac{\text{Total mass used in a process (in kilogram)}}{\text{Mass of final product (in kilogram)}}$$
Mass productivity (MP)	Mass productivity (%) = MI ×100
Reaction Mass Efficiency (RME)	$$RME = \frac{1}{1+E_m}$$ *where E_m is a value of E-factor based on mass*
Atom Utilization (AU)	$$MI = \frac{mass\ of\ the\ final\ product}{total\ mass\ of\ all\ the\ substances\ produced} \times 100$$
Solvent and catalyst environmental impact parameter (f)	$$f = \frac{\sum mass\ of\ reaction\ and\ postreaction\ solventsand\ materials + mass\ [illegible]}{mass\ of\ final\ product}$$

Table S2. Calculated metrics for the oxidation of ethane to acetic acid.

Entry	Solvent	Catalyst	E-factor theoretical	E-factor	Atom Econ. %	Mass intensity	Mass productivity %	Reaction Mass Efficiency (RME)	Atom utilization %	Solvent and catalyst environmental impact parameter (f)
1		$[ReCl_2\{\eta^2\text{-N,O-C(O)Ph}\}(PPh_3)_2]$	0.4	20.8	20.0	444.4	0.2	4.6	100.0	3403.3
2		$[ReCl_2\{N_2C(O)Ph\}(Hpz)(PPh_3)_2]$	0.5	18.9	20.0	396.8	0.3	5.0	100.0	3035.8
3		$[ReCl_2\{N_2C(O)Ph\}(Hpz)_2(PPh_3)]$	0.4	1.9	44.7	58.6	1.7	35.0	96.5	465.9
4		$[ReOCl_3(PPh_3)_2]$	0.4	4.0	44.7	82.7	1.2	20.0	79.1	802.1
5		$[ReCl_3\{HC(pz)_3\}]$	0.2	11.2	20.0	285.6	0.4	8.2	100.0	2200.2
6		$[ReO_3\{\text{-}SO_3C(pz)_3\}]$	0.3	1.2	44.7	50.0	2.0	44.5	95.8	402.2
7	TFA	$[ReClF\{N_2C(O)Ph\}(Hpz)_2(PPh_3)]$	0.2	0.5	44.7	18.9	5.3	66.9	86.0	165.2
8		$Na_2[MoO_4]$	0.1	1.2	20.0	58.0	1.7	45.5	100.0	448.6
9		Nb_2O_5	0.3	2.5	20.0	158.4	0.6	28.7	100.0	1243.4
10		H4[PMo11VO40] × 34H2O	0.9	1.6	44.7	23.4	4.3	39.1	91.3	187.0
11		H5[PMo10V2O40] × 32H2O	0.8	3.5	44.7	38.4	2.6	22.1	83.7	336.0
12		H6[PMo9V3O40] × 34H2O	0.8	1.6	44.7	23.9	4.2	38.6	90.8	192.8
13		C24H19Cl2NO3PRe	0.1	10.0	44.7	71.7	1.4	9.1	75.7	680.5
14	TFA	$C_{21}H_{32}N_2AuCl$ (**1**)	0.2	2.7	20.0	90.3	1.1	26.9	100.0	696.8
15	ACN/H_2O		0.2	0.5	20.0	36.5	2.7	66.5	100.0	107.1
16	TFA	$C_{21}H_{30}N_2AuCl$ (**2**)	0.2	1.9	20.0	71.6	1.4	34.0	100.0	552.6
17	ACN/H_2O		0.2	0.7	20.0	41.0	2.4	59.4	100.0	120.1
18	TFA	$C_{21}H_{30}N_2OAuCl$ (**3**)	0.2	4.0	20.0	120.2	0.8	20.1	100.0	927.2
19	ACN/H_2O		0.2	0.8	20.0	42.4	2.4	57.1	100.0	124.3
20	TFA	$C_{27}H_{44}N_2AuCl$ (**4**)	0.2	2.9	20.0	91.6	1.1	25.9	100.0	705.7
21	ACN/H_2O		0.2	0.8	20.0	42.9	2.3	55.3	100.0	125.8

Table S3. Calculated frequency metrics for the oxidation of ethane to acetic acid.

Entry	Solvent	Catalyst	Time /h	e-factor frequency	Atom Economy over time /%	Mass intensity	Mass productivity /%	Reaction Mass Efficiency Frequency (RMEF) /%	Atom utilization over time /%	Solvent and catalyst environmental impact frequency parameter (ft)
1	TFA	$[ReCl_2\{\eta^2\text{-}N,O\text{-}C(O)Ph\}(PPh_3)_2]$	20	1	1	444	0	0	5	170
2		$[ReCl_2\{N_2C(O)Ph\}(Hpz)(PPh_3)_2]$	20	1	1	397	0	0	5	152
3		$[ReCl_2\{N_2C(O)Ph\}(Hpz)_2(PPh_3)]$	20	0	2	59	2	2	5	23
4		$[ReOCl_3(PPh_3)_2]$	20	0	2	83	1	1	4	40
5		$[ReCl_3\{HC(pz)_3\}]$	20	1	1	286	0	0	5	110
6		$[ReO_3\{\text{-}SO_3C(pz)_3\}]$	20	0	2	50	2	2	5	20
7		$[ReClF\{N_2C(O)Ph\}(Hpz)_2(PPh_3)]$	20	0	2	19	5	3	4	8
8		$Na_2[MoO_4]$	20	0	1	58	2	2	5	22
9		Nb_2O_5	20	0	1	158	1	1	5	62
10		H4[PMo11VO40] × 34H2O	20	0	2	23	4	2	5	9
11		H5[PMo10V2O40] × 32H2O	20	0	2	38	3	1	4	17
12		H6[PMo9V3O40] × 34H2O	20	0	2	24	4	2	5	10
13		C24H19Cl2NO3PRe	20	0	2	72	1	0	4	34
14	TFA	$C_{21}H_{32}N_2AuCl$ (**1**)	20	0	1	90	1	1	5	35
15	ACN/H_2O		20	0	1	37	3	3	5	5
16	TFA	$C_{21}H_{30}N_2AuCl$ (**2**)	20	0	1	72	1	2	5	28
17	ACN/H_2O		20	0	1	41	2	3	5	6
18	TFA	$C_{21}H_{30}N_2OAuCl$ (**3**)	20	0	1	120	1	1	5	46
19	ACN/H_2O		20	0	1	42	2	3	5	6
20	TFA	$C_{27}H_{44}N_2AuCl$ (**4**)	20	0	1	92	1	1	5	35
21	ACN/H_2O		20	0	1	43	2	3	5	6
